# The idiosyncratic drug-induced gene expression changes in HepG2 cells

**DOI:** 10.1016/j.dib.2017.07.074

**Published:** 2017-07-29

**Authors:** J. Jiang, K. Mathijs, L. Timmermans, S.M. Claessen, A. Hecka, J. Weusten, R. Peters, J.H. van Delft, J.C.S. Kleinjans, D.G.J. Jennen, T.M. de Kok

**Affiliations:** a Department of Toxicogenomics, GROW School for Oncology and Developmental Biology, Maastricht University, Maastricht, The Netherlands; b DSM Resolve, Geleen, The Netherlands; c van’t Hoff Institute for Molecular Science (HIMS), Universiteit van Amsterdam, Amsterdam, The Netherlands; d DSM Coating Resins, Waalwijk, The Netherlands

## Abstract

The inflammatory stress has been associated with an increase in susceptibility to idiosyncratic drug-induced liver injury (DILI). However, the molecular mechanisms of this inflammation-associated idiosyncratic drug hepatotoxicity remain unknown. We exposed HepG2 cells with high and low doses of three idiosyncratic (I) and three non-idiosyncratic (N) compounds, in the presence (I+ and N+) or absence (I− and N−) of a cytokine mix for 6, 12 and 24 h. To investigate the genome‐wide expression patterns, microarray was performed using the Agilent 4×44K Whole Human Genome chips. The data presented in this DIB include the expression of genes participating in the ceramide metabolism, ER stress, apoptosis and cell survival pathways. The functions of these genes were illustrated in our associated article (Jiang et al., 2017) [1]. Raw and normalized gene expression data are available through NCBI GEO (accession number GSE102006).

**Specifications Table**

**Table:** 

Subject area	*Biology*
More specific subject area	*(hepato)toxicogenomics*
Type of data	*Table, figure (Venn diagram)*
How data was acquired	*Agilent 4x44K Whole Human Genome microarray*
Data format	*Differentially expressed gene(DEG) tables, Venn diagram, quantile normalization, statistical analysis*
Experimental factors	*HepG2 cells were exposed to high and low doses of three known idiosyncratic (I) and three non-idiosyncratic (N) drug, in the presence (I+ and N+)or absence (I*− *and N*−*)of a cytokine mix for 6, 12 and 24 h.*
Experimental features	*The I+ or N+-exposed samples were corrected for the DMSO+ control.*
	*The I*−*or N*− *-treated groups were compared to the DMSO*− *control.*
Data source location	*Department of Toxicogenomics, Maastricht University, the Netherlands*
Data accessibility	*Microarray data are available through NCBI GEO (accession number GSE102006).*

**Value of the data**

• The cell model and the approaches used in this study can serve as a benchmark to investigate the effects of other idiosyncratic compounds.
• The DEGs obtained in the exposed HepG2 cells can serve as reference points for other studies related to the inflammation-mediated DILI which are conducted using other cell types.
• The listed gene can be used in functional analysis and facilitate the development of the biomarker-based screening tools for the early detection of DILI.

## 1 Data

**Fig. 1:**
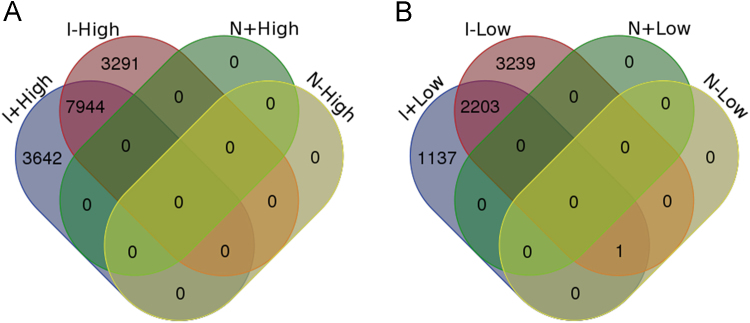
Venn diagram of combined differently expressed gene lists after treated HepG2 cells with high- (A) or low-dose (B) I+, I−, N+ and N−. The diagram shows the number of genes significantly modulated by the different treatments.

Microarray analyses showed that the expression of more than 11,000 genes was significantly changed after exposure to the high-dose I compounds, regardless the presence of the cytokine mix. Around 30% of these DEGs represented the treatment-specific effects. At low-dose, several thousands of DEGs were identified when treated with the I compounds with or without cytokines. Among these genes, over 1000 and 3000 DEGs were unique to the I+ or I− exposures. The N compounds did not have any overt effects on gene expression in the HepG2 cells. A general overview of the DEGs of each treatment is presented in the Venn diagram (Fig. 1). The results of the functional annotation of these treatment-specific DEGs indicated that both the high and low doses of I+ resulted in the immune response-associated BP-GO terms. However, the “apoptosis and survival” associated pathways only appeared after the high-dose I+ treatment. Pathway analysis suggested that the mechanisms, including ceramide metabolism, ER stress, NF-kB signaling and mitochondria activities, are involved in the high-dose I+ induced apoptosis. The FC and the significance of the DEGs involved in these pathways are presented in Table 1, Table 2, Table 3. A more detailed description of these results can be found in Jiang et al. [1].

**Table 1:** The high-dose treatment-induced fold changes of genes involved in TNF signaling, apoptosis pathways and ceramide metabolism (6 h).

Gene symbol	High-dose induced fold change 6 h			
	I+	I−	N+	N−
TNF and apoptosis pathway				
ATF6	**1.34**	−1.02	−1.04	1.12
BAD	−**1.38**	−**1.37**	−1.19	−1.11
BAX	−**1.62**	***−1.39***	−1.19	−1.15
BCL2	1.06	1.16	−1.28	1.04
BCL2L11	1.11	1.08	1.76	1.05
CASP3	**1.39**	1.19	1.04	−1.06
CASP4	***1.23***	**−1.30**	1.09	1.05
CYCS	−1.34	***−1.56***	1.07	−1.05
CHOP/DDIT3	**3.49**	**1.99**	−1.04	1.10
ERN1	**1.59**	**1.82**	1.32	1.14
FAS	−1.05	1.04	1.10	1.02
MAP2K4	**1.24**	**1.28**	1.06	1.08
MAP3K1	**1.32**	1.16	−1.08	−1.07
MAP3K14	1.07	1.32	1.26	1.25
MAPK8	**1.30**	**1.66**	1.04	1.02
NFKB1	**1.38**	**1.41**	1.24	1.21
NFKB2	−1.14	1.22	1.09	1.02
TNF	**5.14**	**2.60**	1.28	1.07
TNFRSF1A	**1.29**	**1.47**	1.11	1.10
TNFSF13B	**1.32**	**1.41**	1.32	1.09
TRAF2	1.09	−1.10	−1.02	−1.10
TRAF6	**1.82**	**2.34**	1.14	1.09

Ceramide metabolism				
**ASAH1**	1.05	−1.09	1.03	1.02
**ASAH2**	1.20	**1.35**	1.07	−1.13
**ASAHL/NAAA**	1.29	−1.10	1.03	−1.05
**CERS6**	−1.13	−1.09	1.05	1.06
**SGMS1**	−1.13	**−1.31**	1.05	1.06
**SGMS2**	**4.43**	**4.82**	1.28	1.58
**SGPP1**	**1.62**	1.06	−1.02	1.12
**SMPD1**	−1.03	−1.17	1.05	−1.24
**SMPD2**	**−1.61**	***−1.28***	−1.06	1.02
**SMPD3**	−1.15	−1.30	−1.22	1.00
**SPHK1**	**−1.37**	***−1.24***	−1.03	−1.12
**SPHK2**	**1.39**	−1.19	1.19	−1.04
**SPTLC1**	−1.07	1.19	1.14	1.23
**SPTLC2**	**1.40**	***1.23***	−1.07	1.02

**Bold** values significant at |FC|>1.5, *p*<0.01 and FDR<0.01 and **Bold** values significant at |FC|**>**1.2, *p*<0.01 and FDR<0.01, values in ***italic*** significant at |FC|>1.2, *p*<0.05.

**Table 2:** The high-dose treatment-induced fold changes of genes involved in TNF signaling, apoptosis pathways and ceramide metabolism (12 h).

Gene symbol	High-dose induced fold change 12 h			
	I+	I−	N+	N−
TNF and apoptosis pathway				
ATF6	**1.42**	1.10	1.14	1.10
BAD	**−1.43**	**−1.30**	−1.04	1.04
BAX	**−1.87**	**−1.52**	1.04	1.04
BCL2	1.49	1.29	1.30	1.23
BCL2L11	−1.05	***−1.33***	−1.03	−1.23
CASP3	**1.34**	**1.27**	−1.05	−1.09
CASP4	**1.32**	**1.35**	***1.33***	1.03
CYCS	−1.33	−1.45	−1.11	−1.03
CHOP/DDIT3	**3.58**	*1.52*	1.15	1.05
ERN1	**2.23**	**2.19**	−1.08	1.01
FAS	−1.05	−1.03	−1.04	−1.10
MAP2K4	**1.46**	**1.49**	1.10	1.07
MAP3K1	−1.02	1.13	−1.12	−1.04
MAP3K14	**1.23**	1.16	1.16	1.10
MAPK8	**1.64**	**1.47**	1.10	−1.05
NFKB1	**1.85**	**1.33**	***1.26***	−1.02
NFKB2	**1.77**	**1.46**	***1.47***	1.04
TNF	**12.23**	1.26	1.83	−1.21
TNFRSF1A	**1.44**	**1.76**	1.13	1.19
TNFSF13B	**1.39**	**1.41**	1.01	−1.10
TRAF2	***1.31***	−1.04	1.16	−1.04
TRAF6	**1.91**	**2.11**	1.04	1.01

Ceramide metabolism				
**ASAH1**	**−1.42**	−1.17	−1.03	−1.03
**ASAH2**	1.12	***1.50***	−1.25	1.19
**ASAHL/NAAA**	**−1.59**	**−1.38**	−1.02	−1.05
**CERS6**	***−1.28***	**−1.38**	1.04	−1.08
**SGMS1**	**−1.24**	−1.13	−1.04	−1.01
**SGMS2**	**3.48**	**2.51**	1.13	−1.02
**SGPP1**	**1.58**	**1.42**	1.13	1.04
**SMPD1**	**−1.46**	−1.27	−1.23	1.05
**SMPD2**	**−1.61**	−1.02	1.13	1.19
**SMPD3**	**−1.81**	**−1.77**	−1.04	−1.15
**SPHK1**	−1.19	1.02	−1.01	−1.01
**SPHK2**	1.01	−1.05	−1.08	1.04
**SPTLC1**	1.05	1.25	−1.20	1.06
**SPTLC2**	1.20	***1.29***	−1.06	−1.12

**Bold** values significant at |FC|>1.5, *p*<0.01 and FDR<0.01 and **Bold** values significant at |FC|>1.2, *p*<0.01 and FDR<0.01, values in ***italic*** significant at |FC|>1.2, *p*<0.05.

**Table 3:** The high-dose treatment-induced fold changes of genes involved in TNF signaling, apoptosis pathways and ceramide metabolism (24 h).

Gene symbol	High-dose induced fold change 24 h			
	I+	I−	N+	N−
TNF and apoptosis pathway				
ATF6	**1.33**	1.05	−1.04	1.03
BAD	−1.04	**−1.34**	1.10	−1.06
BAX	***−1.62***	−1.21	1.33	−1.05
BCL2	1.19	1.29	1.24	1.47
BCL2L11	**2.38**	1.08	1.44	−1.02
CASP3	**1.37**	1.15	−1.04	−1.15
CASP4	1.22	1.14	−1.03	1.13
CYCS	−1.21	−1.16	−1.16	−1.07
CHOP/DDIT3	**3.80**	**2.55**	1.04	−1.07
ERN1	**3.07**	**3.31**	−1.01	1.17
FAS	**1.58**	1.19	−1.01	1.06
MAP2K4	**1.38**	**1.29**	1.02	−1.12
MAP3K1	***1.30***	1.19	−1.07	1.10
MAP3K14	**1.68**	1.02	−1.02	−1.03
MAPK8	**1.52**	**1.40**	−1.02	−1.04
NFKB1	***1.33***	1.11	−1.15	1.06
NFKB2	**1.31**	1.21	−1.11	1.29
TNF	**4.29**	1.84	−1.04	1.22
TNFRSF1A	**1.56**	**1.81**	−1.08	−1.02
TNFSF13B	**1.89**	**1.68**	1.06	1.02
TRAF2	**1.22**	−1.20	1.05	−1.08
TRAF6	**1.73**	**1.82**	1.04	1.01

Ceramide metabolism				
**ASAH1**	**−1.54**	1.01	−1.01	−1.07
**ASAH2**	**1.48**	**1.29**	1.03	−1.05
**ASAHL/NAAA**	**−1.45**	−1.15	1.08	−1.09
**CERS6**	**1.31**	1.03	−1.10	1.11
**SGMS1**	**1.28**	1.17	−1.09	−1.11
**SGMS2**	**3.34**	**2.89**	−1.26	1.01
**SGPP1**	**2.00**	**1.50**	1.07	1.23
**SMPD1**	−1.02	**−1.46**	−1.01	−1.09
**SMPD2**	1.14	1.03	1.08	−1.06
**SMPD3**	−1.27	**−2.39**	−1.19	−1.19
**SPHK1**	−1.07	−1.23	−1.12	−1.09
**SPHK2**	**−1.48**	−1.03	1.08	1.02
**SPTLC1**	**2.25**	**2.21**	1.07	−1.01
**SPTLC2**	***1.30***	1.18	−1.04	1.08

**Bold** values significant at |FC|>1.5, *p*< 0.01 and FDR<0.01 and **Bold** values significant at |FC|>1.2, *p*<0.01 and FDR<0.01, values in ***italic*** significant at |FC|>1.2, *p*<0.05.

## 2 Experimental design, materials and methods

### 2.1 Compound selection and cytotoxicity tests

Three pairs of known idiosyncratic drugs (Nimesulide, Nefazodone, and Trovafloxacin) with their non-idiosyncratic “comparison” drugs (Aspirin, Buspirone, and Levofloxacin) were selected based on the publication of Cosgrove et al. [2]. The main criterion for selecting these compounds was the difference in cytotoxicity between incubations with and without the addition of cytokines, assuming the strongest idiosyncratic effects in those compounds showing the largest difference.

### 2.2 Cell culture and treatment

HepG2 cells were cultured in T25 flasks in the presence of minimal essential medium supplemented with 1% nonessential amino acids, 1% sodium pyruvate, 2% penicillin/streptomycin, and 10% fetal bovine serum (all from Gibco BRL, Breda, The Netherlands) at 37 °C in a humidified chamber with 95%/5% air/CO2. After reaching 80% confluence, the culture medium was replaced with fresh medium containing one of the selected idiosyncratic (I) and non-idiosyncratic (N) compounds, or vehicle control with (+) or without (−) the co-treatment of a cytokine mix as described previously [2] (TNF 100 ng/ml+IFNγ 100 ng/ml+IL-1α 20 ng/ml+IL-6 20 ng/ml+LPS 10 µg/ml). Cells were incubated for 6, 12 or 24 h before being used for gene expression analysis. Three independent biological experiments were conducted for each treatment condition.

### 2.3 RNA isolation

Total RNA was isolated using QIAzol reagent with the RNeasy kit according to the manufacturer's protocol. RNA concentrations were measured on a nanodrop spectrophotometer and the integrity was determined with a bio-analyzer (Agilent Technologies, The Netherlands). Only samples with a good quality (clear 18S and 28S peaks and the RNA integrity number>6) were used for hybridization. Extracted RNA was stored at −80 °C until it was used as the template for cDNA synthesis.

### 2.4 Microarray preparation and data validation

Samples were labeled with Cyanine 3 (Cy3) following the Agilent one-color Quick-Amp labeling protocol (Agilent Technologies, Amstelveen, The Netherlands) and applied on the Agilent 4×44K Whole Human Genome microarray. Then, samples were hybridized and washed according to Agilent's manual. The chips were scanned using the Agilent Microarray Scanner (AgilentTechnologies, Amstelveen, The Netherlands). Raw data on pixel intensities were extracted from the scan images using the Agilent Feature Extraction Software (Agilent). In this study, 252 samples were collected. All chips were checked for their quality using ArrayQC (https://github.com/BiGCAT-UM/arrayQC_Module/), a quality control pipeline in R (version 2.10.1; The R Foundation for Statistical Computing, Vienna, Austria). For each spot the following steps were taken: local background correction, flagging of bad spots, controls and spots with too low intensity, log2 transformation, and quantile normalization. Probes with less than 30% flagged bad spots were selected, missing values were imputed by k-nearest neighbors (k-NN; *k*-value 15) and repeated identifiers merged. Subsequently, the remaining 28796 probes, representing 15163 unique known genes, were used for statistical analysis.

### 2.5 The selection of DEGs and the treatment-specific genes

Gene expression data were log2 transformed. The I+ or N+-exposed samples were corrected for the DMSO+ control and the I−or N− -treated groups were compared to the DMSO- control per dose (low and high) and per time point (6, 12 and 24 h). A change was considered significant when the following criteria were fulfilled: (1) a false discovery rate-corrected *p*-value<0.05 and (2) a log2 fold change of ≤−0.58 or >0.58 (absolute fold change >1.5) for the average of the three replicates. Venn diagrams (Fig. 1) were used to determine the DEGs that were unique to I+, I−, N+ and N− treatments at high- and low-dose, respectively.

### 2.6 Significant gene ontology (GO) terms

The biological process-related gene ontology (BP-GO) terms of treatment-specific DEGs were determined using ConsensusPathDB (CPDB) (http://cpdb.molgen.mpg.de/). The 15163 unique known genes from the 28796 probes were used as background. The GO terms (level 3) describing biological processes with *p*-value<0.05 and FDR<0.05 were considered significant. The significant GO terms containing the keywords “immune”, “response to”, “stimulus”, “stress” and “inflammat*” were considered as relevant. The DEGs involving in the immune response and stimulus-associated GO terms were combined based on different treatment groups.

### 2.7 Pathway analysis of the immune response- and stimulus-related DEGs

MetaCore™ (Thomson Reuters, http://thomsonreuters.com/metacore/) to assess the functional enrichment of the immune response- and stimulus-related DEGs. Pathways with *p*-value<0.05 and FDR<0.05 were considered significant. In this study, expression data of genes involved in the significant pathways related to ‘apoptosis and survival’ are available in Table 1, Table 2, Table 3.

## Conflict of Interest

None.
